# Identification of Rat Ventral Tegmental Area GABAergic Neurons

**DOI:** 10.1371/journal.pone.0042365

**Published:** 2012-07-31

**Authors:** Elyssa B. Margolis, Brian Toy, Patricia Himmels, Marisela Morales, Howard L. Fields

**Affiliations:** 1 Ernest Gallo Clinic and Research Center, University of California San Francisco, Emeryville, California, United States of America; 2 Department of Neurology, University of California San Francisco, San Francisco, California, United States of America; 3 Wheeler Center for the Neurobiology of Addiction, University of California San Francisco, San Francisco, California, United States of America; 4 Cellular Neurophysiology, National Institute on Drug Abuse, Baltimore, Maryland, United States of America; Institute for Interdisciplinary Neuroscience, France

## Abstract

The canonical two neuron model of opioid reward posits that mu opioid receptor (MOR) activation produces reward by disinhibiting midbrain ventral tegmental area (VTA) dopamine neurons through inhibition of local GABAergic interneurons. Although indirect evidence supports the neural circuit postulated by this model, its validity has been called into question by growing evidence for VTA neuronal heterogeneity and the recent demonstration that MOR agonists inhibit GABAergic terminals in the VTA arising from extrinsic neurons. In addition, VTA MOR reward can be dopamine-independent. To directly test the assumption that MOR activation directly inhibits local GABAergic neurons, we investigated the properties of rat VTA GABA neurons directly identified with either immunocytochemistry for GABA or GAD65/67, or *in situ* hybridization for GAD65/67 mRNA. Utilizing co-labeling with an antibody for the neural marker NeuN and *in situ* hybridization against GAD65/67, we found that 23±3% of VTA neurons are GAD65/67(+). In contrast to the assumptions of the two neuron model, VTA GABAergic neurons are heterogeneous, both physiologically and pharmacologically. Importantly, only 7/13 confirmed VTA GABA neurons were inhibited by the MOR selective agonist DAMGO. Interestingly, all confirmed VTA GABA neurons were insensitive to the GABA_B_ receptor agonist baclofen (0/6 inhibited), while all confirmed dopamine neurons were inhibited (19/19). The heterogeneity of opioid responses we found in VTA GABAergic neurons, and the fact that GABA terminals arising from neurons outside the VTA are inhibited by MOR agonists, make further studies essential to determine the local circuit mechanisms underlying VTA MOR reward.

## Introduction

The essential role of the VTA in the motivational and reinforcing actions of MOR agonists is well established [1]–[6], however the local circuit mechanisms are uncertain. Because MOR agonists in the VTA increase both dopamine release in the ventral striatum [7]–[9] and the firing of putative VTA dopamine neurons [10]–[12], and because dopamine contributes to the motivational actions of a variety of natural and drug rewards, the idea that activation of VTA dopamine neurons is required for opioid reward has been widely accepted.

While local MOR agonists do activate some VTA dopamine neurons, the direct synaptic effects of opioid receptor activation are typically inhibitory. The canonical two neuron model of opioid reward proposes that, as in other brain regions [13], MOR excites midbrain VTA dopamine neurons indirectly by hyperpolarizing local GABAergic interneurons [14], [15]. However, in the original studies VTA neurons were ‘identified’ as GABAergic if they were directly inhibited by a MOR agonist; clearly this is circular reasoning when testing the hypothesis that MOR agonists work by inhibiting GABA release [15], [16]. Conversely, VTA neurons were ‘identified’ as dopaminergic if they were inhibited by dopamine D2 receptor activation but not MOR activation. Subsequent research has demonstrated that the neurons of the VTA are considerably more diverse than had been assumed by Johnson & North [15], [16]. For example, contrary to the canonical model, a significant proportion of cytochemically identified dopamine neurons are directly inhibited by MOR agonists [17]–[19]. Further complicating the issue is the recent discovery that, in addition to GABAergic and dopaminergic neurons, the rat VTA has a significant population of glutamatergic neurons; this clearly challenges another critical assumption of the canonical model [16], i.e. that all non-dopamine neurons in the VTA are GABAergic [20], [21]. Finally, and most challenging for the model, dopamine is not required for MOR agonist reward in mouse [22] or rat [23]–[25]. Therefore, while MOR agonists acting in the VTA are both necessary and sufficient to produce positive reinforcement, the synaptic and local circuit mechanisms for this action are uncertain.

A recent study in the mouse supports the canonical two neuron model [26]. Almost all mouse VTA neurons were reported to be either dopaminergic or GABAergic, and, in contrast to the rat and other mouse studies, MOR agonists inhibited all GABAergic but no dopamine neurons. The homogeneity of these two neuronal groups is exactly what was assumed when the first *ex vivo* characterizations of dopamine and putative GABAergic VTA neurons were completed [16] and provides critical support for Johnson and North's proposal that postsynaptic inhibition of VTA GABAergic interneurons locally connected to dopamine neurons mediates MOR reward [15]. On the other hand, in the rat, it is clear that dopamine neurons are pharmacologically heterogeneous and significant numbers of neurons are neither dopamine nor GABA containing. Since most *in vivo* studies of MOR reward have been carried out in rat, it is essential to examine the properties of rat VTA GABA neurons to relate synaptic actions of VTA MOR to behavior. While inhibitory MOR responses have been shown in identified rat VTA GABA neurons, this was done in a limited sample consisting solely of projection neurons having unknown local connections [27]. If, in fact, there is heterogeneity of GABAergic local connectivity and the MOR response of GABA neurons is not uniform, alternative models for VTA opioid reward must be considered. For example, there is robust presynaptic inhibition of GABA release onto VTA neurons by MOR agonists [18], [28]–[30]. Although these GABAergic terminals could arise from local interneurons [31], several brain regions give rise to GABAergic inputs to the VTA [32]–[38], and GABA release at these terminals is inhibited by MOR agonists [39], [40].

To systematically and directly examine the validity of the canonical two neuron model of the VTA in the rat, we made *ex vivo* recordings in VTA neurons and used post hoc immunocytochemical and *in situ* hybridization methods to directly identify GABA neurons. We compared the physiological and pharmacological properties of VTA GABA neurons to those neurons that were negative for GABA markers, as well as to immunocytochemically identified dopamine neurons.

## Results

### Anatomy

**Figure 1:**
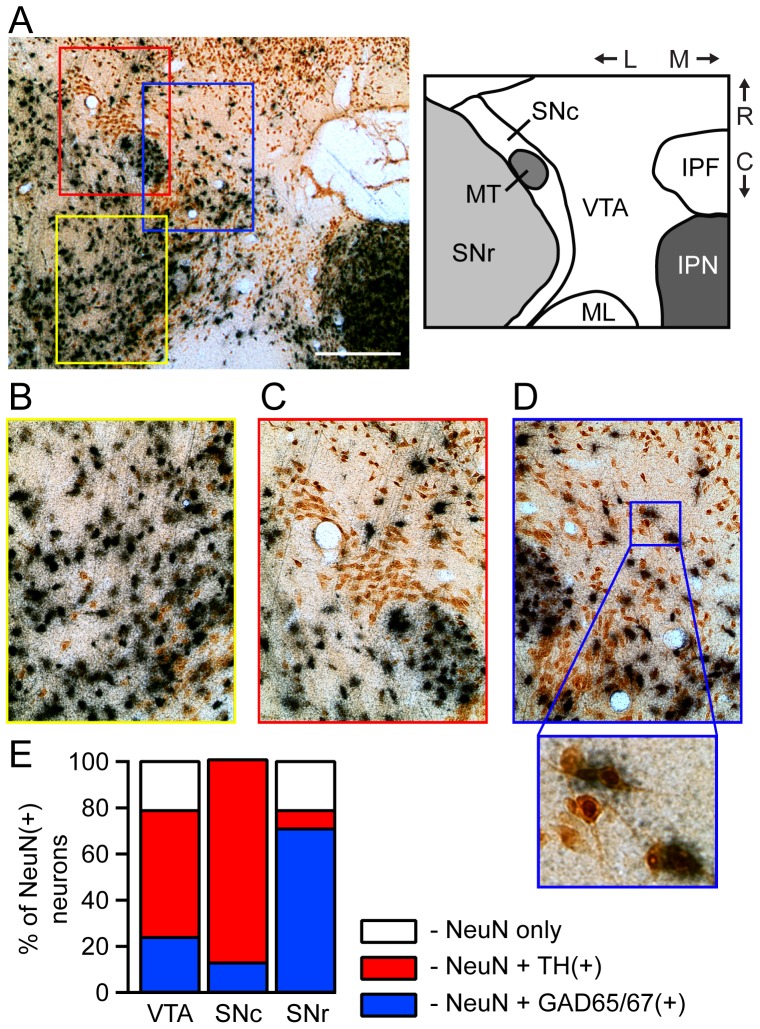
Mean density of GABA neurons in VTA: approximately 20% are GABAergic. In horizontal brain slices containing the VTA and the SN the general neural marker NeuN was cytochemically visualized with the secondary label DAB (brown); GABAergic neurons were labeled using *in situ* hybridization against GAD65/67 mRNA (black grains). (A, left) Low magnification example image of one of the slices used to estimate the percentage of neurons that are GABAergic. Scale bar: 400 µm. Schematic (right) illustrates slice orientation and anatomical landmarks. (IPF: interpeduncular fossa; IPN: interpeduncular nucleus; ML: medial lemniscus; MT: medial terminal nucleus of the accessory optic track; SNc: substantia nigra pars compacta; SNr: substantia nigra pars reticulata; R: rostral; C: caudal; M: medial; L: lateral). Higher magnification images (color coded rectangles show location in (A)) show GAD65/67(+) and GAD65/67(−) in the SNr (B - yellow border), the SNc (C – red border), and the VTA (D – blue border with higher magnification section below). (E), Mean percentage of GAD65/67(+) neurons in VTA, SNc, and SNr neurons and, for comparison, our prior estimates of % TH(+) neurons [41].

To unambiguously assess the percentage of VTA neurons that are GABAergic, we first co-labeled horizontal VTA slices using *in situ* hybridization against mRNA for GAD65/67 and an antibody against the nuclear neuronal marker NeuN (Figure 1A). We utilized *in situ* hybridization against GAD65/67 for this counting because we expected this approach to yield the fewest false negative neurons. Using tissue from three animals, twelve representative images were sampled from randomly selected areas throughout the VTA, replicating methods we used to estimate the percentage of VTA neurons that are dopaminergic [41]. All VTA image locations corresponded to anatomical locations where we had previously demonstrated immunoreactivity against tyrosine hydroxylase (TH), a marker of dopamine neurons. An average of 61±4 NeuN(+) neurons were counted per image (range 13–116 neurons). We found that 23±3% of NeuN-labeled neurons in the VTA had a spatially correlated density of grains indicating GAD65/67 mRNA content (Figure 1D). In spite of regional inhomogeneity of % colabeling across VTA images (2%–52%), we did not detect any consistent topographical pattern of GABA neuron density (dorsal: 25±2% vs ventral: 21±5%, *P* = 0.4; rostral: 21±3% vs caudal: 25±2%, *P* = 0.15; medial: 21±2% vs lateral: 26±3%, *P* = 0.14). GAD labeling was also distinct at the VTA side of the border with the substantia nigra pars compacta (SNc), which in many places made this border clearly discernible (Figure 1C,D). Together, these results and our previous estimate of percent of TH(+) VTA neurons leave approximately 22% of VTA neurons that are neither GABAergic or dopaminergic by immunostaining criteria (Figure 1E). One obvious possibility is that these unlabeled neurons are glutamatergic [20].

For comparison with these VTA data, additional images, 3 per region, were analyzed from the SNc and substantia nigra pars reticulata (SNr) from each animal. Consistent with prior reports that the SNc is predominantly dopaminergic (including our own estimate that 88% of SNc neurons are TH(+) [41]), we detected GAD65/67 grain clusters associated with 13±4% of SNc neurons (Figure 1C). In the SNr we found that 71±5% of neurons were GAD65/67 mRNA positive (Figure 1B). This percentage is slightly lower than expected given that we previously found that only 8% of SNr neurons are TH(+) and to date only dopamine and GABA neurons have been found in the SNr (Figure 1E). Post hoc examination of the images from the SNr revealed that the background density of grains in the SNr was higher than in any other region counted. This increase in background grains could obscure the counting of neurons weakly expressing *in situ* signal so that the neurons may have been counted as GAD65/67(−). Adding to this possible undercounting, the dense packing of intensely labeled neurons would make lighter labeling in adjacent neurons more difficult to detect.

### Electrophysiology

**Figure 2:**
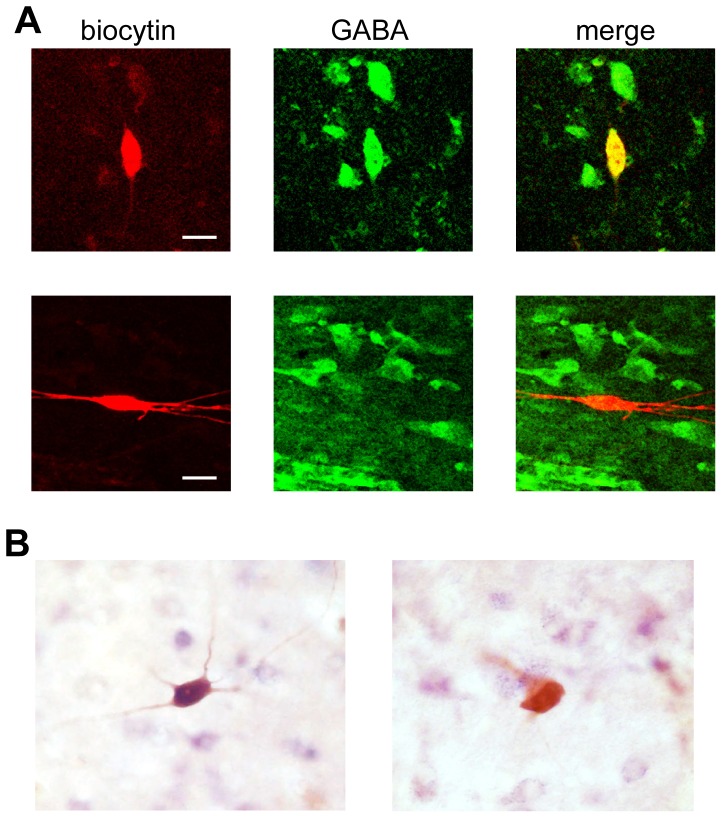
Identification of GABA neurons following whole cell recordings. (A) Example where immunocytochemistry against GABA (green) was used to identify GABAergic VTA neurons filled with biocytin (red). (Top row) An example GABA(+) recorded cell, and (Lower row) an example GABA(−) recorded cell. (B) In other cases we used *in situ* hybridization against GAD65/67 mRNA to test whether recorded neurons were GABAergic. In these examples of positive colabeling (left) and a lack of colabeling (right), filled cells are labeled with DAB (brown), and *in situ* label is visualized with digoxin (purple).

**Figure 3:**
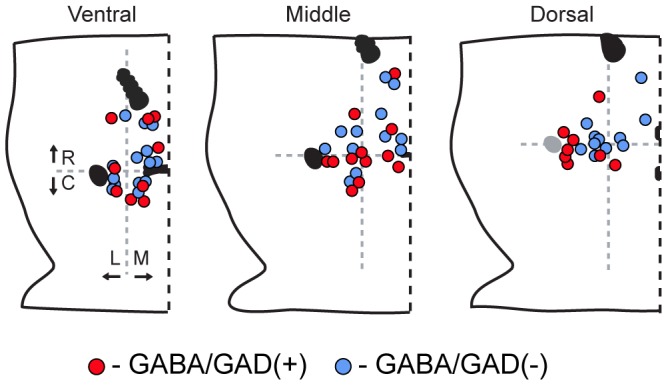
Locations of recorded VTA GABA neurons. Sample includes neurons throughout the VTA. Schematic diagrams of ventral, middle, and dorsal horizontal slices containing the VTA. (R: rostral; C: caudal; M: medial; L: lateral).

Since we previously showed that the size and shape of cytochemically identified GAD67(+) neurons in the VTA are similar to those of TH(+) neurons [41], and because we found that GABA neurons are distributed throughout the VTA, we completed *ex vivo* whole cell recordings in VTA neurons without setting explicit, *a priori* cell selection criteria. In different sets of filled cells, we utilized either immunocytochemistry against GABA (Figure 2A), immunocytochemistry against GAD65/67, or *in situ* hybridization for GAD65/67 mRNA (Figure 2B) to directly identify the neurotransmitter content of electrophysiologically characterized VTA neurons. We recorded from a total of 31 confirmed GABAergic neurons, and the observed properties were consistent across the different *post hoc* procedures. The numbers of neurons identified with each approach reported for each measurement are reported in Table S1. Locations of all recorded GABA neurons are indicated in Figure 3. Recordings were limited to neurons intermixed with A10 dopaminergic neurons.

**Figure 4:**
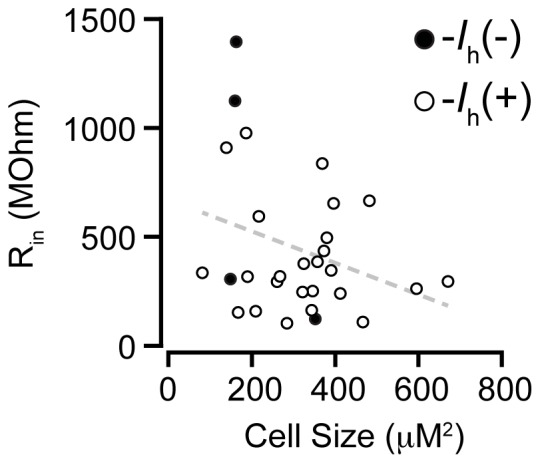
Plot of soma crossectional areas and input resistances for all confirmed GABAergic neurons. There was a trend towards an inverse relationship between these two measures. *P* = 0.09.

Consistent with our previous anatomical findings [41], the recorded neurons confirmed as GABAergic displayed shapes similar to VTA dopamine neurons including fusiform (n = 12), elliptical (n = 7), multipolar (n = 8), and round (n = 2) cells. Confirmed GABA neuron cross-sectional areas included a wide range (81.4–670.6 µm^2^; mean = 312±26 µm^2^; Figure 4) and were also comparable to TH(+) neurons [41]. These findings suggest that our sample of recorded neurons is representative of the range of VTA GABAergic neurons.

The initial membrane potential of VTA GABA neurons was also similar to that of TH(+) neurons (GABA/GAD65/67(+): −45.5±1.8 mV, n = 30; TH(+): −43.3±0.4 mV, n = 233; *P* = 0.22). The distribution of R_in_ in VTA GABA neurons, displayed in Figure 4, was also similar to that of TH(+) neurons (GABA/GAD65/67(+): 460±60 MΩ, n = 30; TH(+): 390±20 MΩ, n = 233; *P* = 0.12) [41].

**Figure 5:**
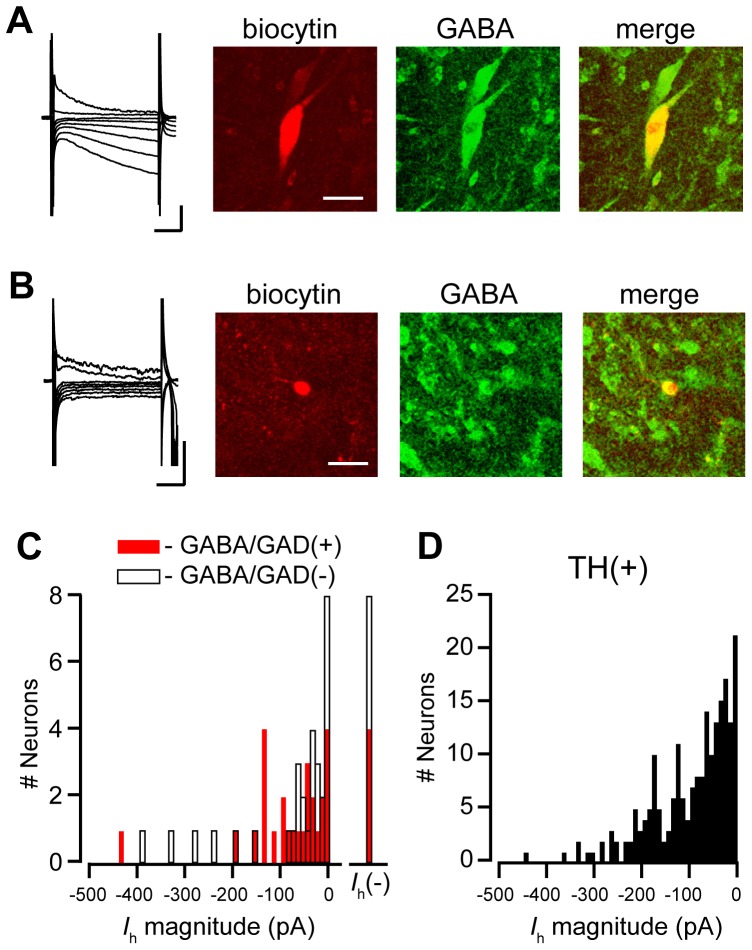
Most VTA GABA neurons are *I*_h_(+). (A) Example GABAergic neuron that has a large *I*_h_ (far left, scale bars: 100 pA and 50 ms). The neuron was filled with biocytin (left; red; scale bar: 20 µm) and immunocytochemically confirmed GABA(+) (middle, green). (B) Example *I*_h_(−) GABAergic neuron (far left, scale bars: 50 pA and 50 ms) that was filled with biocytin (left; scale bar: 20 µm) and confirmed GABA(+). (C) Histogram of *I*_h_ magnitudes measured in GABA/GAD(+) and GABA/GAD(−) neurons. (D) For comparison, histogram of *I*_h_ magnitudes from recorded VTA dopamine neurons identified with TH immunocytochemistry.

**Figure 6:**
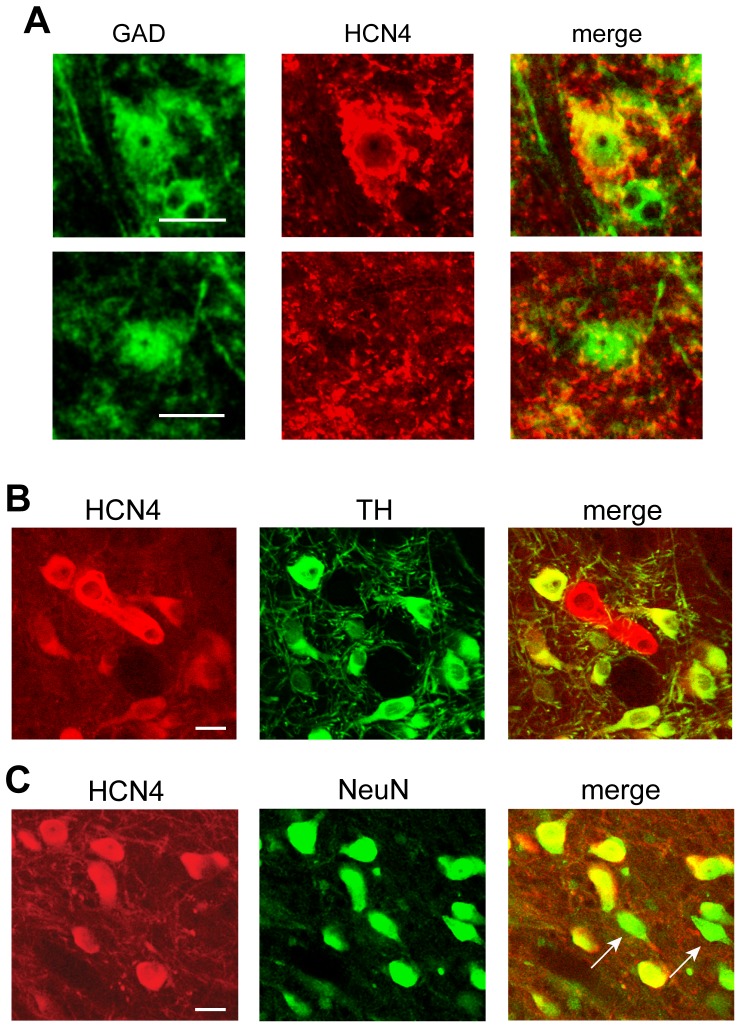
VTA neurons express HCN protein. (A) Immunocytochemical colabeling in VTA tissue for GAD and HCN4, one of the channels expressed in the VTA that produces the *I*_h_, yields examples of GAD(+) neurons that are HCN4(+) (top) or HCN4(−) (bottom), consistent with the physiology. Scale bars: 20 µm. (B) Immunocytochemical colabeling for HCN4 and TH in VTA slices shows that HCN4 is expressed in some TH(−) neurons. Scale bar: 20 µm. (C) The HCN4 antibody was not non-specifically labeling all neurons as HCN4 colabeling with the neural marker NeuN shows that some VTA neurons were cytochemically HCN4(−). Scale bar: 20 µm.

We tested each recorded neuron for the presence of an *I*_h_, the hyperpolarization activated cation current that is commonly used to identify putative dopamine neurons in *ex vivo* recordings. Most confirmed GABA neurons expressed an h current (Figure 5A,C); only 4 out of 31 confirmed GABA neurons were *I*_h_(−). Further, the distribution of amplitudes of the *I*_h_ in confirmed GABA neurons was not noticeably different from that of TH(+) neurons (Figure 5C,D). These data are consistent with our previous finding that many TH(−) neurons express an *I*_h_ [41]. To provide further evidence that VTA GABA neurons express an *I*_h_, we completed immunocytochemical experiments using an antibody against HCN4, one of the channel subunits responsible for the *I*_h_ expressed in the VTA [42], [43]. We found that HCN4 was expressed in many VTA neurons, including GAD65/67(+) neurons (Figure 6A). In separate tissue we found TH(−) neurons that were HCN4(+) (Figure 6B). Finally, to demonstrate that the HCN4 antibody was not indiscriminately labeling all neurons, we labeled slices for both HCN4 protein and NeuN. We found a small population of NeuN(+) VTA neurons that were not labeled with the HCN4 antibody, consistent with the small percentage of *I*_h_(−) neurons we find physiologically with wide sampling of VTA neurons (Figure 6C) [41].

**Figure 7:**
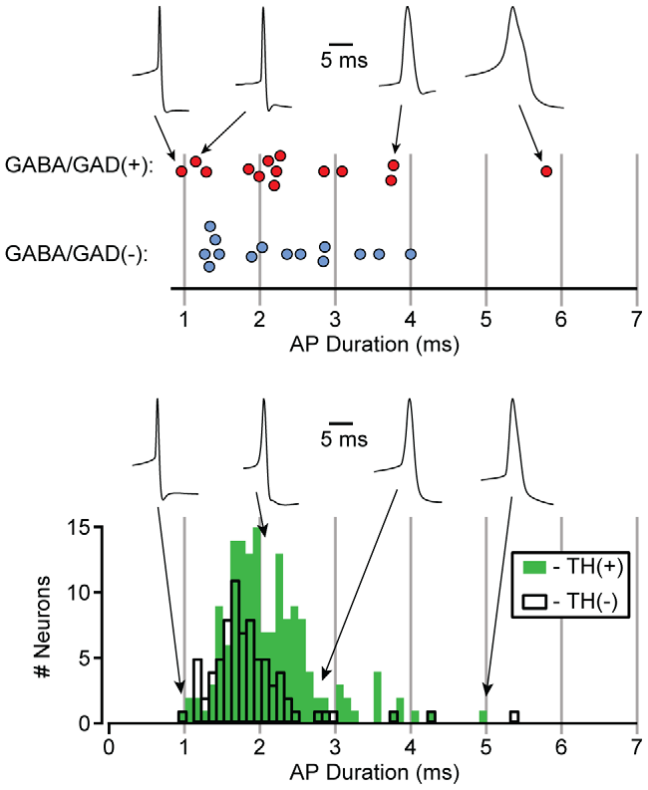
The distribution of action potential durations in VTA GABA neurons overlaps with that of cytochemically confirmed dopamine neurons. Neurons recorded on the same day were identified post hoc as GABA/GAD(+) or GABA/GAD(−), and their AP duration distributions are overlapping (top). This is consistent with data from TH(+) and TH(−) neurons where the range AP durations of TH(−) neurons spans the range for TH(+) neurons (bottom).

Although action potential (AP) duration has been used to identify VTA GABA neurons, we previously showed that both TH(+) and TH(−) neurons have a broad range of AP durations, raising the possibility that some GABA neuron AP durations are longer than previously assumed. We measured AP durations in neurons that were spontaneously firing immediately after gaining whole cell access. We observed a wide distribution of AP durations in confirmed VTA GABA neurons. Their AP durations overlapped with those of both GABA/GAD65/67(−) neurons recorded on the same days as the GABA/GAD65/67(+) neurons and TH(+) neurons (Figure 7).

**Figure 8:**
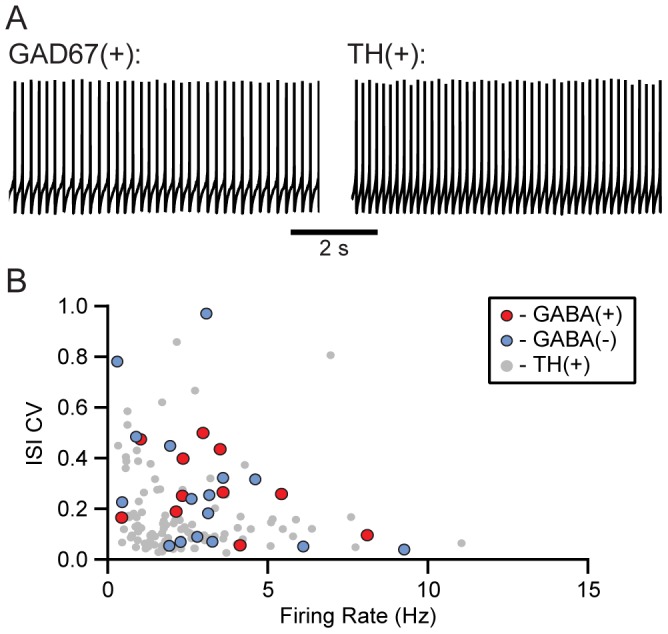
The spontaneous firing rates of VTA GABA neurons are similar to those of dopamine neurons *ex vivo*. (A) Example traces of AP activity from a confirmed VTA GABA neuron (left) and a confirmed dopamine neuron (right) firing at similar frequencies. (B) For each spontaneously firing neuron, the ISI CV is plotted against the firing rate of the neuron.

It has been asserted that VTA neurons that have faster firing rates are GABAergic. For instance, Johnson and North [16] reported that their secondary cells, or putative GABA neurons, were mostly either quiescent or fired faster than their principal, putative dopamine, neurons. Of our confirmed GABA neurons, 11 were firing spontaneously (35%). This is not significantly different from TH(+) neurons, where 117/261 (45%) were spontaneously firing (*P* = 0.35, Fisher exact test). Many of the identified GABA neurons recorded in this study had slow spontaneous firing rates similar to those observed in confirmed dopamine neurons (Figure 8), and the population distribution was not different from that of the confirmed dopamine neurons (GABA/GAD65/67(+): 3.3±0.6 Hz, n = 11; TH(+): 2.3±0.2 Hz, n = 117; *P* = 0.1). We also analyzed the coefficient of variation of the interspike intervals (ISI CV) of confirmed GABA and TH(+) neurons to evaluate the regularity of the pacemaker firing in the different populations of neurons. While the range of ISI CVs was similar for TH(+) and GABA/GAD65/67(+) neurons, there was a difference in the distributions of ISI CVs in relation to firing rate. Specifically, in neurons with firing rates below 3.35 Hz, the ISI CV is significantly greater in GABA/GAD65/67(+) neurons compared to TH(+) neurons (GABA/GAD65/67(+): 0.63±0.24; TH(+): 0.21±0.02; *P* = 0.00004; Figure 8B). Therefore, among neurons with similarly low spontaneous firing rates *ex vivo*, there is a subset of VTA dopamine neurons whose firing is more regular than VTA GABA neurons.

Although our sample is limited, we analyzed whether there were differences between the *I*_h_(+) and *I*_h_(−) VTA GABA neurons. There was a trend for *I*_h_(−) neurons to be smaller (*P* = 0.098) with higher R_in_ (*P* = 0.051) compared to the *I*_h_(+) GABA neurons (Figure 4, Figure 5A,B). There was no difference in V_mi_ between *I*_h_(+) and *I*_h_(−) neurons (−46±2 mV, −39±4 mV, respectively, *P* = 0.18). Two of the *I*_h_(−) GABA neurons were firing spontaneously, and consistent with our previous work, the intracellular AP durations of the *I*_h_(−) neurons overlapped those of *I*_h_(+) neurons (3.00±0.78 ms and 2.44±0.59 ms, respectively). The firing rates of the two spontaneously active *I*_h_(−) neurons were also no different from those of the *I*_h_(+) GABA neurons (*I*_h_(−): 3.25±0.27 Hz; *I*_h_(+): 3.28±0.79 Hz). Together, these data demonstrate that there are minimal physiological differences that sort in GABA neurons according to their *I*_h_ expression.

### Pharmacology

**Figure 9:**
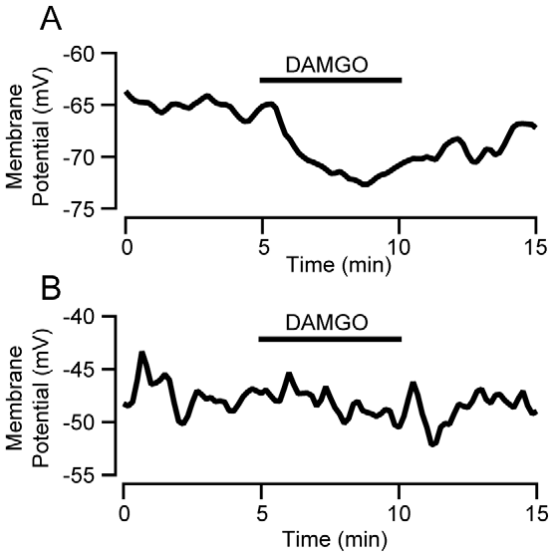
Some, but not all, VTA GABA neurons are inhibited by activation of the MOR. Example current clamp traces from identified VTA GABA neurons tested with bath application of 1 µM DAMGO. (A) GABA neuron showing robust inhibition by DAMGO (B) GABA neuron unresponsive to DAMGO.

Central to the canonical two neuron model are the responses of VTA dopamine and GABA neurons to either MOR or dopamine D2 receptor activation. Specifically, the model asserts that VTA GABA neurons are inhibited by MOR activation but insensitive to D2 receptor agonists. We found that the MOR agonist DAMGO (1 µM or 500 nM) inhibited 7 out of 13 directly identified GABA/GAD65/67(+) neurons (Figure 9). Therefore, while some VTA GABA neurons are indeed inhibited by MOR activation, a significant proportion is not. Together with the multiple demonstrations of MOR inhibition of dopamine neurons [17]–[19], the lack of MOR agonist response in nearly half of GABAergic neurons renders MOR agonist inhibition completely unreliable for identification of a VTA neuron as GABAergic in the rat.

The other pharmacological response proposed to sort by neural types in the VTA is the dopamine D2 “autoreceptor” (D2R) inhibition in putative dopamine, but not GABA, VTA neurons. We previously demonstrated that many confirmed dopamine neurons do not show a hyperpolarization in response to the D2R agonist quinpirole (1 µM) [41], [44]. In fact, this property varies with projection target: many, but not all, confirmed dopamine neurons that project to the nucleus accumbens and medial prefrontal cortex are inhibited by quinpirole, while none of the dopamine neurons projecting to the amygdala were inhibited by quinpirole [44]. Among all VTA neurons we have confirmed as TH(+), 32/53 (60%) were significantly inhibited by quinpirole. Further, among TH(−) neurons, 14/47 (30%) were inhibited by quinpirole. This suggests that some GABA neurons could be inhibited by quinpirole. In fact, two out of 4 GABA/GAD65/67(+) neurons tested with quinpirole were hyperpolarized, confirming that some VTA GABA neurons are in fact inhibited by D2R activation. It is interesting to note that our pharmacological data matches the data of Johnson and North [16]. That is, they reported that the vast majority of VTA neurons were inhibited by either MOR activation or D2R activation, but not both. In our data, neither of the quinpirole-inhibited neurons responded to DAMGO, but the two quinpirole non-responsive neurons were inhibited by DAMGO. These findings add to the growing evidence for pharmacologic heterogeneity among VTA neurons, even within a subgroup defined by neurotransmitter content.

**Figure 10:**
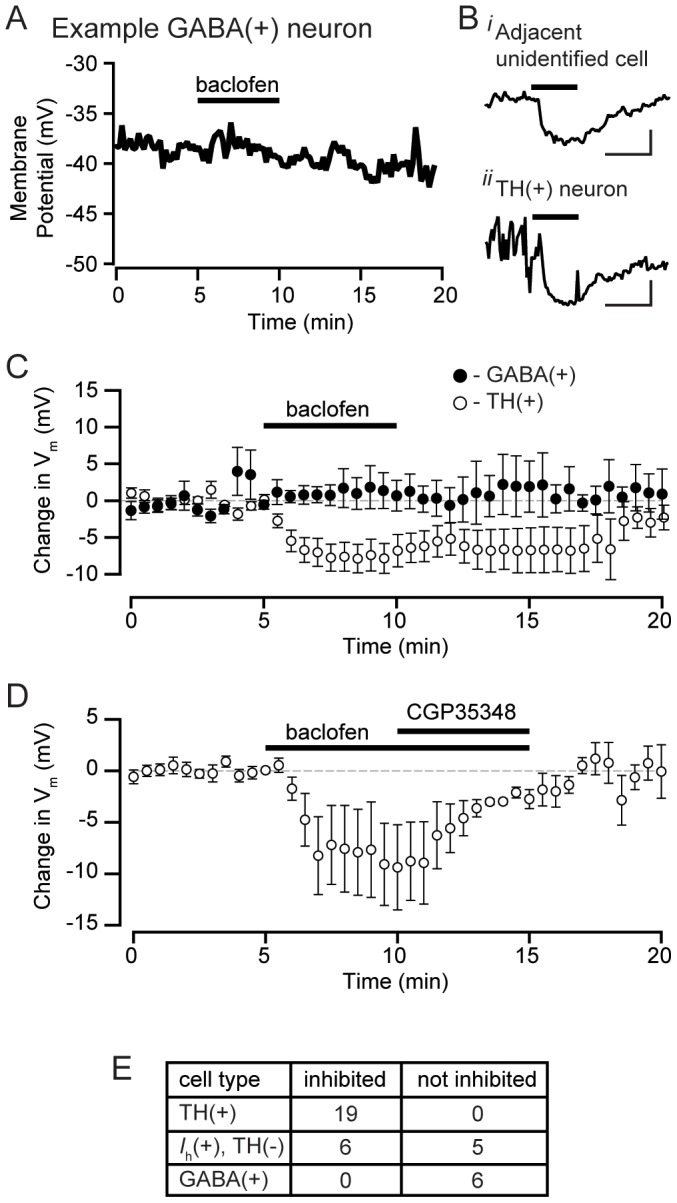
GABA_B_ receptor activation inhibits VTA dopamine neurons, but not VTA GABA neurons. (A) Example current clamp traces from cytochemically identified GABA neuron lacking a response to the GABA_B_ receptor agonist baclofen (1 µM). (B*i*) An unidentified neuron recorded on the same day as GABA(+) neurons that were insensitive to baclofen shows a significant hyperpolarization in response to baclofen (1 µM; bar). (B*ii*) Example of a cytochemically identified dopamine neuron hyperpolarized by baclofen (1 µM; bar). Scale bars: 5 mV and 5 min. (C) Average trace of identified GABA neurons (n = 6) tested with bath application of baclofen (1 µM) compared to the averages of all quiescent confirmed dopamine neurons (n = 8). (D) Average trace of 4 neurons significantly inhibited by baclofen (1 µM); the inhibition was reversed by the GABA_B_ receptor antagonist CGP35348 (10 µM). (E) Table of all observed baclofen responses across confirmed GABA, dopamine, and *I*_h_(+), TH(−) neurons. TH-identified neurons include spontaneously firing, quiescent (from panel C), and voltage-clamp experiments.

Previous investigators have proposed models of VTA GABA function that have GABA_B_ receptors located specifically on dopamine neurons and not on local GABA interneurons [42], [43], [45], [46]. Cytochemical studies have also raised the possibility that VTA GABA_B_ receptors are localized mainly to TH(+) neurons [47]. However, GABA_B_ receptor agonist responses have never been tested using electrophysiology in confirmed VTA GABA neurons. Interestingly, no GABA/GAD65/67(+) neurons (0/6) were hyperpolarized by the GABA_B_ receptor agonist baclofen (1 µM; Figure 10A,C). Neurons that were not GABA/GAD65/67(+) but recorded on the same experimental days as the GABA/GAD65/67(+) neurons often responded to baclofen (Figure 10B*i*). Further, 19/19 confirmed TH(+) neurons were sensitive to baclofen (Figure 10B*ii*,C,E). Baclofen induced hyperpolarizations were also reversed by the GABA_B_ receptor antagonist CGP35348 (10 µM; Figure 10 D). Therefore, there is a highly significant difference in the expression of functional GABA_B_ receptors on VTA GABA neurons compared to VTA dopamine neurons (*P*<0.00001, Fisher exact test), making this property the most reliable pharmacological difference between GABAergic and dopaminergic VTA populations. However, it is important to note that we have observed baclofen induced inhibitions in non-dopamine neurons as well. Specifically, 6/11 *I*_h_(+), TH(−) neurons were significantly inhibited by baclofen, while 2/6 *I*_h_(−) neurons were inhibited. Since GABA neurons are not inhibited, by exclusion this finding suggests that most, if not all, VTA glutamate neurons are inhibited by GABA_B_ receptor activation. Such an effect is unlikely to be a critical confound for dopamine neuron identification in the lateral VTA where glutamate neurons are sparse, but may require serious consideration in the medial VTA where there is a high density of glutamate neurons [20], [48].

## Discussion

We have demonstrated that VTA GABAergic neurons are heterogeneous with respect to their physiologic and pharmacologic properties in the rat. While some VTA GABA neurons have properties consistent with the assumptions of the canonical two neuron model, a large proportion do not. Therefore alternative synaptic and local circuit mechanisms for VTA MOR agonist reward must be seriously entertained.

### The previously assumed properties of GABAergic VTA neurons in the rat

Defining rat VTA secondary cells as those inhibited by MOR and not by dopamine D2 receptor agonists, Johnson and North [16] reported additional properties of this subpopulation of neurons that they postulated were GABAergic interneurons. Compared to VTA principal, putative dopamine neurons, secondary cells had, on average, shorter duration APs. Johnson and North also found that some secondary neurons fired faster than any principal neurons, sometimes in bursts, and only 43% of their secondary cells expressed an h current, compared to all principal neurons. However, it is critical to recognize that these data describe MOR-inhibited VTA neurons, not necessarily GABA neurons, and subsequent work has repeatedly shown that many cytochemically confirmed dopamine neurons in rat VTA are inhibited by MOR agonists [17]–[19]. On the other hand, one subset of rat VTA GABAergic neurons does appear to have consistent inhibitory responses to MOR activation: Steffensen et al. [49] described a population of midbrain neurons with uniform physiological properties whose anatomical distribution overlaps with the ventral tier of the red nucleus as well as the A10 VTA dopamine neurons. Those neurons had high firing rates, very short duration APs, and were antidromically activated by stimulation of the internal capsule. They were confirmed as GABA-containing by ultrastructure cytochemistry [49], and were inhibited by MOR activation [27]. Although these VTA GABA projection neurons are inhibited by MOR agonists, there is no direct evidence that those neurons contribute to the local inhibition of dopamine neurons, as required by the canonical model. Furthermore, since we show that there are subpopulations of VTA GABA neurons with different physiologic and pharmacologic properties, including a significant number that are not postsynaptically inhibited by MOR agonists, one cannot conclude that inhibition of local GABA neurons is either necessary or sufficient for MOR activation of dopaminergic neurons.

### Characteristics of confirmed dopaminergic and GABAergic rat VTA neurons

Consistent with the requirements of the two neuron canonical model for VTA MOR reward we demonstrate here that some GABAergic neurons in the rat VTA are inhibited by the MOR selective agonist DAMGO. However, in contrast to the assumptions of Johnson and North in the rat and Chieng and colleagues' report in mouse [26], the properties of rat VTA GABA neurons are heterogeneous and overlap extensively with those of cytochemically identified VTA dopamine neurons. Rat VTA GABA neurons often express large *I*_h_s, long duration APs, and slow spontaneous firing rates, making them physiologically indistinguishable from VTA TH(+) neurons. Furthermore, we also found that a significant subset of VTA GABA neurons is not inhibited by MOR activation.

The one feature we found that does distinguish rat VTA GABA and dopamine neurons is their sensitivity to the GABA_B_ receptor agonist baclofen. VTA GABA neurons lack a response to GABA_B_ receptor activation, compared to TH(+) neurons where every neuron we tested was inhibited by GABA_B_ receptor activation. This is similar with prior studies in rat and mouse VTA where much smaller responses were seen to baclofen in putative GABA neurons compared to putative dopamine neurons [50]. However, the neurotransmitter content of these neurons was predicted by *I*_h_ expression, making it difficult to relate to the mostly *I*_h_(+) identified GABA neurons described here, especially in view of the fact that only a third of our *I*_h_(−) neurons were confirmed GABA/GAD65/67(+). It is of interest that while we have observed no TH(+), *I*_h_(−) neurons in rat VTA, we have seen some *I*_h_(−) neurons that are also GABA/GAD65/67(−). The most likely reason for this is that at least some VTA glutamate neurons are *I*_h_(−). Further, immunocytochemical results [47] and our own observations of baclofen inhibitions in TH(−) and *I*_h_(−) neurons (unpublished data) also suggest that some VTA glutamate neurons are inhibited by GABA_B_ receptor activation. However, since glutamate neurons are sparse in the lateral VTA, there are likely some VTA subregions where baclofen responses are strong predictors of dopamine neural content. In fact, given our current data and the distribution of VTA glutamate neurons, baclofen responses are likely to yield the lowest combination of false negative and false positive identification of VTA dopamine neurons of any tool to date.

The insensitivity of GABA neurons to baclofen is also interesting in light of VTA microinjection studies where GABA_B_ receptor agonists often produce different behavioral outcomes than GABA_A_ receptor agonists, [e.g.51], [52]. Our data also suggest that using baclofen to inactivate the VTA, [e.g.53], will primarily if not exclusively inhibit VTA dopamine neuron activity while potentially leaving GABAergic signaling intact. Therefore, this pharmacological difference between VTA GABA and other neurons could be a useful identification tool as well as a method for relatively selective manipulation of non-GABAergic VTA neurons.

### Properties of GABA and DA neurons in the VTA of the mouse

Recently, Chieng et al. [26] utilized GAD-GFP mice to identify GABA neurons and putative dopamine neurons in the VTA. In contrast to our findings in rat, they report that the responses of GFP(+) identified GABA neurons were homogeneous; all were inhibited by MOR agonists but not dopamine. Importantly, their cytochemical analysis suggests that only 2% of VTA neurons were neither GABAergic (GAD-GFP(+)) or dopaminergic (TH(+)), consistent with the assumptions of Johnson and North in rat (1992b) but contrary to the data presented here; consequently, they could assume that GFP(−) neurons were overwhelmingly dopaminergic. Consistent with Johnson and North's report in the rat [16], their GFP(−) neurons were inhibited by dopamine, but not MOR agonists. Also consistent with Johnson and North, Chieng et al. reported that GFP(+) neurons had shorter duration APs than GFP(−) neurons. Furthermore, the GFP(+) cells they found expressed very small to absent *I*_h_ while many of the GFP(−) neurons expressed a large *I*_h_. Their findings not only differ from our results in the rat but contrast with other studies showing significant heterogeneity in mouse VTA neurons. For example, Cohen et al. [54] using extracellular recordings found no difference in AP duration between optogenetically identified dopaminergic and GABAergic VTA neurons. Also inconsistent with Chieng et al.'s report that 92% of GAD-GFP(+) VTA neurons were inhibited by MOR activation and none of the GFP(−) neurons were sensitive to MOR, Ford and colleagues [18] reported that amygdala-projecting TH(+) neurons in the mouse are directly inhibited by MOR activation. Another discrepancy is with Lammel et al. [55] who reported that prefrontal cortex-projecting VTA dopamine neurons are not sensitive to dopamine D2 receptor activation, while Chieng et al. report that all GFP(−) neurons are inhibited by dopamine. Similarly, another study of mouse VTA that did not select neurons by their projection target found that 30% of confirmed dopamine neurons were not inhibited by D2 receptor activation (personal communication, T. Hnasko). Regarding whether or not *I*_h_ sorts by neurotransmitter content, Lammel and colleagues reported that some dopamine neurons in the mouse are *I*_h_(−), again inconsistent with Chieng et al.'s report. While it seems likely that different approaches to neuronal sampling in electrophysiological studies may account for some of the quantitative differences in VTA neuronal properties reported from different laboratories, the use of *I*_h_, AP duration, and dopamine 2DR and direct MOR agonist inhibition as markers of neurotransmitter content in mouse VTA neurons should be considered unreliable.

### VTA GABA neuron circuitry and the canonical two neuron model

The canonical two neuron model of VTA circuitry proposes that local GABA neurons synapse onto (and only onto) VTA dopamine neurons, and that the prototypical reward-relevant projection of the dopamine neuron is to the ventral striatum. There is a growing consensus that this is a highly reduced picture of VTA local circuitry, even with regard to the connections of GABAergic neurons. For example, VTA GABA neurons not only make local connections but a significant number project to other brain regions, including prefrontal cortex, ventral striatum, dorsal raphe nucleus, and ventral periaqueductal gray [31], [44], [56]–[58]. Local GABA synapses on to dopamine neurons may arise from local interneurons that lack extra-VTA projection targets or may be collaterals of projecting neurons. Although the canonical model predicts that VTA GABA interneurons express MOR on their somas, it is not clear whether the VTA GABA neurons we found to be inhibited directly by MOR agonists give rise to local connections.

One attractive alternative is that instead of inhibiting the somadendritic region of GABAergic interneurons, MOR agonists disinhibit dopamine neurons by an action on the terminals of GABA axons. In fact, MOR agonists can inhibit over 75% of synaptic GABA release in the VTA through receptors located on GABA terminals [28], [29], [39], therefore disinhibition of dopamine neurons by MOR activation could occur primarily as a result of activation of receptors on presynaptic terminals [14]. Such presynaptic inhibition could control the terminals of local GABA neurons and/or GABA terminals arising from neurons in other brain regions. While GABAergic inputs arising from local neurons and synapsing onto VTA dopamine neurons have been shown anatomically [31] and physiologically [59], [60], GABAergic terminals in the VTA also arise from neurons in the nucleus accumbens, ventral pallidum, and the rostromedial tegmental nucleus (RMTg) [32], [33], [37], [38]. Although inputs from the nucleus accumbens in the rat synapse largely onto non-dopamine VTA neurons [40], inputs from the RMTg [61], ventral pallidum (personal communication, G. Hjelmstad), and LDT [35] synapse onto VTA dopamine neurons. Further, inputs from the RMTg onto VTA dopamine neurons are strongly inhibited by MORs on the axon terminals of these inputs [39]. Therefore, it may be that the disinhibitory effect of MOR agonists within the VTA is due primarily to inhibition of GABA release from terminals of extrinsic rather than local GABA neurons.

There are potentially important mechanistic differences between a model where MOR presynaptically inhibits GABA release onto dopamine neurons compared to the canonical model where local GABA neurons are inhibited postsynaptically. First, for disinhibition to occur in the canonical model, the VTA GABA neurons must be firing so that activating MOR can inhibit the neural activity. Therefore, these GABA neurons must either be spontaneously active or be firing in response to excitatory inputs so that MOR induced hyperpolarizations can decrease activity. In contrast, if disinhibition were due to inhibition of GABA release at terminals, it could be produced in the absence of action potential driven GABA release by decreasing the probability of activity independent spontaneous vesicle fusion. Another significant difference between MOR hyperpolarizing GABA interneurons compared to inhibiting neurotransmitter release at terminals is that when MOR is functional at the soma, activation decreases firing activity to all terminals. In contrast, if MOR activation specifically inhibits GABA release at terminals, only those terminals near the endogenous opioid release site will be affected. Therefore, if a VTA GABA neuron synapses onto topographically dispersed VTA neurons, or has both local collaterals and projections to other brain regions, it is possible that opioid peptide release will not be synchronized across all terminals. Further, it is possible that different terminals of the same neuron differentially express MOR, as a variety of synaptic properties are determined by the postsynaptic cell [62]–[64]. As the data show that MOR strongly inhibits GABA release at terminals, including terminals arising from some extrinsic sources, and only half of our identified VTA GABA neurons were hyperpolarized by DAMGO, it is possible that the characteristics of MOR modulatory influences on axon terminals play a major role in the VTA microcircuitry underlying reward.

Another question that is largely unanswered is what role VTA GABA projection neurons play in behavior, in particular MOR-mediated behavior. We have found many non-dopamine projection neurons that are inhibited by MOR activation (Dopamine 50 Years Congress abs, BO-21, p. 38, 2007; INRC abs, p. 80, 2009), and there is a specific subpopulation of VTA GABA projection neurons that are inhibited by MOR [27]. Such VTA GABA projection neurons could play a role in behavioral responses to VTA MOR, including reward, that are independent of dopamine neurotransmission [23].

### Conclusion

The canonical two neuron model of the VTA provided a simple and elegant model that led to a series of very informative experiments. While some subsequent results were consistent with the model, it is now clear that dopamine neurons are more heterogeneous in their pharmacology and physiology than originally proposed, and that MORs act at several synaptic targets controlling both GABA and dopamine neurons within the VTA. Here we extend this understanding by demonstrating that the properties of VTA GABA neurons are also diverse. They include a wide range of pharmacological responses, AP durations, and firing rates, as well as both *I*_h_(+) and *I*_h_(−) neurons, and these properties overlap to a great extent with those of confirmed dopamine VTA neurons. Importantly, while MOR and D2 receptor agonist responses do not differentiate VTA dopamine and GABA neurons, here we show that dopamine, but not GABA, neurons are inhibited by GABA_B_ receptor activation. These data, together with accumulating evidence that the properties of VTA neurons depend greatly upon their projection target [18], [44], [55], [65] and that VTA-mediated reward is often dopamine-independent, indicate that an expanded concept of the synaptic and local circuit mechanisms of VTA MOR reward is necessary.

## Materials and Methods

All animal protocols were conducted under National Institutes Health (NIH).

Guidelines using the NIH handbook Animals in Research and were approved by the Institutional Animal Care and Use Committee (Ernest Gallo Clinic and Research Center, University of California at San Francisco, Emeryville, CA), approval ID 10.01.202.

### Slice preparation and electrophysiology

Male Sprague-Dawley rats, 22–50 days old, were anaesthetized with isoflurane, decapitated, and the brains were removed. Horizontal brain slices (150 µm thick for immunocytochemistry, 200 µm for *in situ* hybridization) containing the VTA were prepared using a vibratome (Leica Instruments, Germany). The ventral-dorsal landmarks for VTA slice selection were the interpeduncular fossa (IPF) and the linear nucleus of the raphe. Slices were submerged in aCSF containing (mM): 119 NaCl, 2.5 KCl, 1.3 MgSO_4_, 1.0 NaH_2_PO_4_, 2.5 CaCl_2_, 26.2 NaHCO_3_, and 11 glucose, saturated with 95% O_2_–5% CO_2_ and allowed to equilibrate at 35°C for at least 1 h.

Individual slices were visualized under a Zeiss Axioskop with differential interference contrast optics and infrared illumination, using a Zeiss Axiocam MRm and Axiovision 4 software. Whole-cell patch clamp recordings were made at 31°C using 2.5–4 MΩ pipettes containing (mM): 123 potassium gluconate, 10 (*4-(2-hydroxyethyl)-1-piperazineethanesulfonic acid* (HEPES), 0.2 EGTA, 8 NaCl, 2 MgATP, and 0.3 Na_3_GTP (pH 7.2, osmolarity adjusted to 275). Biocytin (0.1%) or Lucifer Yellow (0.05%) was added to the internal solution in order to mark the recorded neuron for later cytochemical characterization. Recordings were made using an Axopatch 1-D (Axon Instruments, Union City, CA, USA), filtered at 2 kHz and collected at 5 kHz or or filtered at 5 kHz and collected at 20 kHz using IGOR Pro (Wavemetrics, Lake Oswego, OR, USA). Liquid junction potentials were not corrected during current- or voltage-clamp recordings. *I*_h_ was measured by voltage clamping cells and stepping from −60 to −40, −50, −70, −80, −90, −100, −110 and −120 mV. Input resistance was monitored with hyperpolarizing pulses while holding the cell in current clamp mode (current set to 0 pA). Data was collected continuously when cells were firing spontaneously or the membrane potential was sampled for a 2 second period once every 10 sec in quiescent neurons.

Quinpirole, [D-Ala^2^, N-Me-Phe^4^, Gly-ol^5^]-Enkephalin (DAMGO) or baclofen were applied by bath perfusion. Stock solution was made and diluted in aCSF immediately prior to application. Quinpirole stock (1 mM) and DAMGO stock (5 µM) were dissolved in H_2_O. Baclofen stock (10 mM) was mixed in water with the pH adjusted with 37% HCl until fully dissolved. Salts and drugs were obtained from Sigma Chemical (St Louis, MO, USA).

### Cytochemistry

For anatomical studies, rats were perfused transcardially with 0.9% saline followed by 4% paraformaldehyde (v/v). Brains were removed and postfixed for 2 h in the same fixative and then stored at 4°C in PBS (pH 7.4) for 24 h. Horizontal brain slices (50 µm thick) containing the VTA were prepared using a vibratome. Every second slice was cytochemically processed. Slices were rinsed with PBS, then pre-blocked for 2 h at room temperature in PBS with 0.3% (v/v) Tween20, 0.2% (m/v) bovine serum albumin (BSA) and 5% (v/v) normal goat serum, and then agitated for 48 h at 4°C with one or more of the following: mouse anti-neuronal nuclei (NeuN) monoclonal antibody (1∶1000, Millipore Corporation), rat sodium/potassium hyperpolarization-activated cyclic nucleotide-gated ion channel 4 monoclonal antibody (1∶500, Thermo Scientific Pierce), rabbit anti-sodium/potassium hyperpolarization-activated cyclic nucleotide-gated ion channel 2 (HCN2) polyclonal antibody (1∶100, Thermo Scientific Pierce), rabbit polyclonal anti-tyrosine hydroxylase (TH) antibody (1∶100, Millipore Corporation), or mouse anti-glutamic acid decarboxylase 67 kDa isoform (GAD67) monoclonal antibody (1∶100, Millipore Corporation). Slices were then rinsed and incubated overnight at 4°C with Cy3 goat anti-mouse, FITC goat anti-rat, and/or Cy5 goat anti-rabbit secondary antibodies.

Slices used for electrophysiology and immunocytochemically processed for TH or GABA expression were fixed immediately after recording in 4% formaldehyde for 2 h. Slices were processed as described above with either TH or rabbit GABA polyclonal antibody (1∶150, Thermo Scientific Pierce, PA1-18027; 1∶200, Abcam Inc., ab8891), followed by Texas Red, Cy3 or Cy5 goat anti-rabbit, anti-mouse secondary antibody and DTAF-conjugated Streptavidin (3.25 µL/mL). Slices processed with antibody ab8891 were fixed in a 2% glutaraldehyde, 2% formaldehyde solution.

Recording slices for GAD immunocytochemistry were fixed immediately after recording in a phosphate buffered (75 mM KH_2_PO_4_, 85 mM Na_2_HPO_4_) 4% formaldehyde solution with 14% picric acid, pH 6.9, for 1 h. These slices were then rinsed in tris-buffered saline (0.9% NaCl, 6.1 g/L tris base, 0.1% NaN_3_, pH 7.4) followed by a 1∶10 diluted PBS solution in de-ionized water. This diluted PBS solution was used for the remaining protocol steps. Slices were preblocked for 2 h at room temperature with 5% normal horse serum and 5% BSA, agitated for 48 h at 4°C with 1∶1000 mouse anti-GAD primary antibody, rinsed and incubated overnight at 4°C with Alexa Fluor 488 donkey-anti mouse (1∶500, Invitrogen, A-21202) secondary antibody and Cy5-conjugated streptavidin (10 µL/mL).

In all cases, sections were mounted on slides using Bio-Rad Fluoroguard Antifade Reagent mounting media and visualized under a Zeiss LSM 510 META microscope.

Unless otherwise stated, secondary antibodies were acquired from Jackson ImmunoResearch Laboratories, Inc. (West Grove, PA, USA) and working dilutions of 1∶100 were used.

### *In situ* hybridization

To quantify the proportion of VTA neurons that are GABAergic we utilized immunocytochemistry against NeuN and radioactive *in situ* hybridization against GAD65 and GAD67 mRNAs. Briefly, 100 g rats were perfused and brains were removed and fixed as above. To prepare tissue for cryosectioning, brains were transferred sequentially to 12%, 14% and 18% sucrose solutions in PBS. Every second slice was processed. Twenty µm cryosections were incubated for 10 min in PBS containing 0.5% Triton X-100, rinsed twice with PBS, treated with 0.2 n HCl for 10 min, rinsed twice with PBS and then acetylated in 0.25% acetic anhydride in 0.1 m triethanolamine, pH 8.0 for 10 min. Sections were rinsed twice with PBS, postfixed with 4% paraformaldehyde for 10 min and, after a final rinse with PBS, were hybridized for 16 h at 55°C in hybridization buffer (50% formamide; 10% dextran sulfate; 5×Denhardt's solution; 0.62 m NaCl; 50 mM dithiothreitol; 10 mM EDTA; 20 mM PIPES, pH 6.8; 0.2% sodium dodecyl sulfate; 250 mg/mL salmon sperm DNA; 250 mg/mL tRNA) containing [^35^S]- and [^33^P]-labeled single-stranded antisense to GAD65 (nucleotides 1–1758, Accession # NM012563) and GAD67 (nucleotides 1–1782, Accession # NM017007). Sections were treated with 4 mg/mL RNaseA at 37°C for 1 h, washed with 1× SSC, 50% formamide at 55°C for 1 h, and with 0.1× SSC at 68°C for 1 h. After the last SSC wash, sections were rinsed with tris-buffered saline (20 mM Tris HCl, 0.5 M NaCl, pH 8.2).

For NeuN colabeling, sections were then rinsed with PBS and incubated for 1 h in PBS supplemented with 4% BSA and 0.3% Triton X-100. Sections were then incubated with overnight in mouse anti-NeuN monoclonal antibody (1∶1000, Millipore) at 4°C. After rinsing 3×10 min in PBS, sections were processed with an ABC kit (Vector Laboratories, Burlingame, CA). The slices were incubated for 1 h at room temperature in a 1∶200 dilution of the biotinylated secondary antibody, rinsed with PBS, and incubated with avidin biotinylated horseradish peroxidase for 1 h. Sections were rinsed and the peroxidase reaction was then developed with 0.05% 3,3-diaminobenzidine-4 HCl (DAB) and 0.003% hydrogen peroxide (H_2_O_2_). Sections were mounted on coated slides, air dried, dipped in nuclear track emulsion and exposed for several weeks prior to development.

Slices in which filled cells were to be tested for GAD65/67 mRNA labeling were fixed immediately after recordings in RNase free 4% formaldehyde for 24 h, then were rinsed in RNase free PBS. These slices were hybridized with the antisense digoxigenin riboprobes for GAD65 (nucleotides 1–1758, Accession # NM012563) and GAD67 (nucleotides 1–1782, Accession # NM017007) obtained by *in vitro* transcription using digoxigenin-11-UTP labeling mix (Roche), and T3 RNA polymerase. Plasmids that contained GAD65 and GAD67 were generously provided by Dr. Allan Tobin (University of California Los Angeles, Los Angeles, CA). Sections were treated with 4 µg/mL RNaseA at 37°C for 1 h, washed with 1× SSC, 50% formamide at 55°C for 1 h, and with 0.1× SSC at 68°C for 1 h. After the last SSC wash, sections were rinsed with tris-buffered saline (20 mM Tris–HCl, 0.5 M NaCl, pH 8.2). Afterwards, sections were incubated with an alkaline phosphatase-conjugated antibody against digoxigenin (Roche Applied Science) overnight at 4°C; the alkaline phosphatase reaction was developed with nitroblue tetrazolium and 5-bromo-4-chloro-3-indolyl phosphate (Roche Applied Science), yielding a purple reaction product. Biocytin signal was detected with avidin biotinylated horseradish peroxidase for 1 h. Sections were rinsed and the peroxidase reaction was then developed with 0.05% 3,3-diaminobenzidine-4 HCl (DAB) and 0.003% hydrogen peroxide (H_2_O_2_).

### Data analysis

Results are presented as mean ± SEM. Statistical comparisons between groups of neurons were made using one way ANOVAs. *P*<0.05 was required for significance. Some data from TH(+) neurons were previously published in [41], [44], [65], [66].

Cell sizes were measured from images collected with the Zeiss Axioskop using Axiovision 4.0. One GABA(+), one GAD(+), and two GABA(−) neurons were excluded from this analysis because there was blurring of the cell outline due to the depth of the cell in the slice or images were not collected at the time of recording. The membrane potential reported here is the initial potential observed immediately after achieving whole-cell configuration. *I*_h_ magnitude was measured as the difference between the initial capacitative response to a voltage step from −60 to −120 mV and the final current during the same 200 ms step. Neurons were considered *I*_h_(−) if the slope of the I–V curve for hyperpolarizing steps from −60 to −90, −100, −110 and −120 mV was 0. One TH(+) neuron was excluded from Figure 4E because the *I*_h_ exceeded −500 pA, and changing the scale to accommodate this one neuron would have made the GABA neuron data harder to examine. One GABA(+) neuron was excluded from the R_in_ and V_mi_ calculations because data at initial whole cell access for that cell was lost. For all AP duration measurements, at least 10 APs were averaged together to reduce the influence of noise on the measurements. Intracellular AP durations were measured within the first 2 minutes of gaining whole cell access to the cell. The intracellular AP duration was measured from the threshold (when the rate of rise exceeded 5 V/s for data collected at 5 kHz or 10 V/s for data collected at 20 kHz) to the time at which the descending voltage recrossed the threshold following the AP peak. We also measured extracellular AP durations in voltage clamp (V = −60 mV) after achieving an at least 15 GOhm seal. We found a significant relationship between our intracellular AP durations and extracellular AP durations (Figure S1). The reported firing rates are averages of the instantaneous firing rate over at least 2 and up to 10 min usually at the beginning of the experiment. ISI CV was calculated as the SD/mean for 50 ISIs at the beginning of each experiment, excluding delays due to brief hyperpolarizations to monitor input resistance. Firing rates and ISI CVs were only included in the analysis if the firing appeared stable over the duration of the measurement. The statistical significance of drug effects was tested within neurons using Student's unpaired t-test for which data were binned into 30 sec samples, comparing the last 4 min of baseline to the last 4 min of drug application.

### Counting procedures

To estimate the percentage of GABAergic neurons in midbrain regions we counted the number of NeuN-labeled neurons and determined the proportion of those cells that had a spatially correlated density of grains from *in situ* hybridization against GAD65/67. Counting was done in duplicate by two independent counters who were blind to the brain region associated with each image, and the results were averaged between the two quantifications. Imaged locations were selected using a random number generator and the number of images per slice was proportional to the area encompassed by the VTA. The rostral border of the VTA was taken as the level of the caudal tip of the mammillary tract and the lateral border was the medial edge of the medial terminal nucleus of the accessory optic tract (MT). Tissue samples were considered dorsal to the VTA if the MT was not visible.

## Supporting Information

Figure S1 **Intracellular and Extracellular AP durations measured from the same neuron are correlated.** (A), example extracellular AP measured in cell-attached mode (top) and example intracellular AP measured from the same cell in whole cell mode (bottom). (B) Plot of extracellular AP durations vs intracellular AP durations, measured as in (A), shows a significant correlation (*P* = 0.047) between these two measures in VTA neurons.
(TIF)

Table S1 **Physiological properties of VTA GABA neurons were tested across multiple identification methods.**
(DOC)
