# Supplementary figures and images for: Identification of Rat Ventral Tegmental Area GABAergic Neurons
Source: PLoS One. 2012 Jul 31;7(7):e42365. doi: 10.1371/journal.pone.0042365 (PMC3409171; doi:10.1371/journal.pone.0042365)

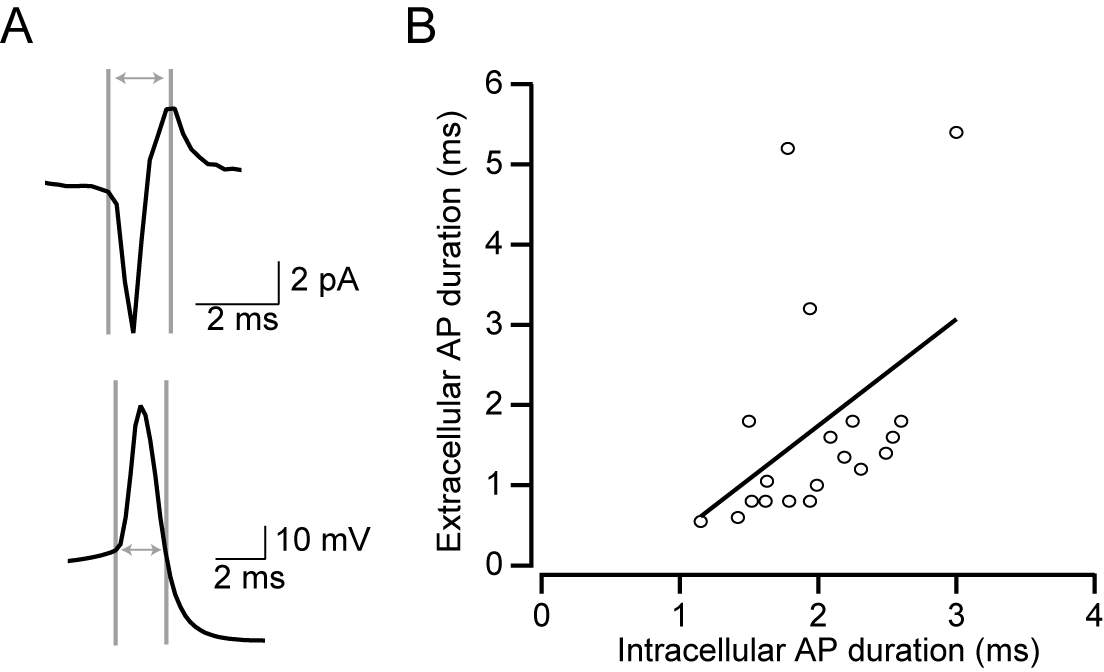

Supplement: Figure S1 — Intracellular and Extracellular AP durations measured from the same neuron are correlated. (A), example extracellular AP measured in cell-attached mode (top) and example intracellular AP measured from the same cell in whole cell mode (bottom). (B) Plot of extracellular AP durations vs intracellular AP durations, measured as in (A), shows a significant correlation (P = 0.047) between these two measures in VTA neurons. (TIF) [file pone.0042365.s001.tif]
