# Supplementary material for: Identification of Rat Ventral Tegmental Area GABAergic Neurons
Source: PLoS One. 2012 Jul 31;7(7):e42365. doi: 10.1371/journal.pone.0042365 (PMC3409171; doi:10.1371/journal.pone.0042365)
Supplement: Table S1 — Physiological properties of VTA GABA neurons were tested across multiple identification methods. (DOC) [file pone.0042365.s002.doc]

| Physiological measurement | # data points from GABA immunocytochemistry | # data points from GAD immunocytochemistry | # data points from GAD in situ hybridization |
| --- | --- | --- | --- |
| R_in_, V_mi_ | 24 | 4 | 2 |
| *I*_h_(+),*I*_h_(-) | 21,4 | 4,0 | 2,0 |
| Intracellular AP duration | 11 | 2 | 1 |
| Firing rate & ISI CV | 6 | 3 | 2 |
| DAMGO | 9 | 2 | 2 |
| Baclofen | 5 | 1 | 0 |
